# TaAMT2;3a, a wheat AMT2-type ammonium transporter, facilitates the infection of stripe rust fungus on wheat

**DOI:** 10.1186/s12870-019-1841-8

**Published:** 2019-06-06

**Authors:** Junpeng Jiang, Jing Zhao, Wanlu Duan, Song Tian, Xiaodong Wang, Hua Zhuang, Jing Fu, Zhensheng Kang

**Affiliations:** 1 State Key Laboratory of Crop Stress Biology for Arid Areas and College of Life Sciences, Northwest A&F University, Yangling, Shaanxi People’s Republic of China; 2 State Key Laboratory of Crop Stress Biology for Arid Areas and College of Plant Protection, Northwest A&F University, Yangling, Shaanxi People’s Republic of China

**Keywords:** *Puccinia striiformis* f.sp*. tritici*, Ammonium transporter, TaAMT2;3a, Stripe rust

## Abstract

**Background:**

Ammonium transporters (AMTs), a family of proteins transporting ammonium salt and its analogues, have been studied in many aspects. Although numerous studies have found that ammonium affects the interaction between plants and pathogens, the role of AMTs remains largely unknown, especially that of the AMT2-type AMTs.

**Results:**

In the present study, we found that the concentration of ammonium in wheat leaves decreased after infection with *Puccinia striiformis* f. sp*. tritici* (*Pst*), the causal agent of stripe rust. Then, an AMT2-type ammonium transporter gene induced by *Pst* was identified and designated as *TaAMT2;3a*. Transient expression assays indicated that TaAMT2;3a was located to the cell and nuclear membranes. TaAMT2;3a successfully complemented the function of a yeast mutant defective in NH_4_^+^ transport, indicating its ammonium transport capacity. Function of *TaAMT2;3a* in wheat-*Pst* interaction was further analyzed by barley stripe mosaic virus (BSMV)-induced gene silencing. *Pst* growth was significantly retarded in *TaAMT2;3a*-knockdown plants, in which ammonium in leaves were shown to be induced at the early stage of infection. Histological observation showed that the hyphal length, the number of hyphal branches and haustorial mother cells decreased in the *TaAMT2;3a* knockdown plants, leading to the impeded growth of rust pathogens.

**Conclusions:**

The results clearly indicate that the induction of AMT2-type ammonium transporter gene *TaAMT2;3a* may facilitates the nitrogen uptake from wheat leaves by *Pst,* thereby contribute to the infection of rust fungi.

**Electronic supplementary material:**

The online version of this article (10.1186/s12870-019-1841-8) contains supplementary material, which is available to authorized users.

## Background

Nitrogen is an indispensable element for all living organisms, and its uptake and utilization are the major limiting factors for plant growth and crop yield [1, 2]. As an essential nutrient, nitrogen is the key component of biological macromolecules such as proteins, nucleic acids, vitamins and hormones. Thus, uptake and utilization of nitrogen affect nearly all biological processes and are tightly regulated [3, 4].

Ammonium and nitrate, which are absorbed and transported into plant root cells by AMTs and NRTs (nitrate transporters) respectively, are the main plant available inorganic forms of nitrogen in soils. Because NO_3_^−^ needs to be reduced to NH_4_^+^ before assimilation in plant cells, NH_4_^+^ is usually more preferentially absorbed than nitrate [1, 5]. After transport into root cells, NH_4_^+^ is assimilated into glutamate via the glutamine synthase/glutamate synthase (GS/GOGAT) cycle for the further synthesis of organic macromolecules.

Plant ammonium transporters belong to multigene families. In *Arabidopsis* and rice, there are 6 and 10 AMTs, respectively, whereas at least 54 AMT homologues are present in the wheat genome [6, 7]. According to their sequence and phylogeny, these AMTs are categorized into two subfamilies, AMT1 and AMT2. AMT1-type AMTs are mainly expressed in roots and play a critical role in ammonium uptake from the soil [8]. Out of the six AMTs in *Arabidopsis*, four AMT1-type transporters contribute to ammonium uptake in root cells. An AMT1;1 AMT1;2 AMT1;3 AMT2;1 quadruple deletion line (*qko*) showed significantly impaired ammonium uptake ability and severe growth depression under ammonium supply [9]. AMT2-type AMTs are usually expressed in various plant tissues, including roots, shoots and leaves at relative low levels. Compared with the well-studied AMT1-type transporters, AMT2-type AMTs are not able to transport ^14^C-Methylamine (an analog of ammonia), which make it difficult to characterize their transport kinetics and physiological roles [10].

It has long been known that the nitrogen status and availability in plants affect the disease process [5]. A considerable number of studies have shown that a high nitrogen level enhances plant susceptibility to biotrophic and hemibiotrophic pathogens, known as nitrogen-induced susceptibility (NIS) [11]. It has been suggested that a high concentration of nitrogen in the host is favourable for nutrient acquisition by pathogens. On the other hand, biotrophic and hemibiotrophic pathogens often attempt to manipulate plant metabolism to their advantage. The induction of host genes related to nitrogen uptake and transport during infection is one of the most common way employed by pathogens. Three wheat ammonium transporter genes, *TaAMT1;1a*, *TaAMT1;1b* and *TaAMT1;3a*, are induced only in compatible interactions between wheat and stem rust fungus [12]. Similarly, the expression of the nitrate transporter gene *NRT2.6* is induced after the inoculation of *Arabidopsis thaliana* by the phytopathogenic bacterium *Erwinia amylovora* [13].

Stripe rust is one of the most serious diseases threatening the growth and production of wheat worldwide. The causal agent, *Puccinia striiformis* f. sp. *tritici* (*Pst*), is a typical obligate biotrophic fungus. The nutrients required for the growth and reproduction of *Pst* come entirely from wheat via haustoria, the special fungal structures formed in the host mesophyll cells [14]. Thus, for rust fungi, uptake of sufficient nutrients is the determinant for successful colonization. Several genes related to sucrose transport and metabolism have been identified and are well documented [15–17]. It has also been demonstrated that leaf nitrogen content is important for sustaining the stripe rust epidemics during winter [18]. Moreover, nitrogen availability in the host leaves significantly affected spore production for wheat leaf rust fungus [19], indicating a direct role of host nitrogen in rust disease. However, it is not well understood how rust fungi manipulate host nitrogen metabolism and acquire nitrogen from the host.

In this study, we analysed the changes in ammonium content in different tissues of wheat infected by stripe rust and identified an AMT2-type wheat gene, *TaAMT2;3a*, induced by *Pst* infection. Then, the subcellular localization of TaAMT2;3a was examined by transient expression of GFP fusion protein, and its ammonium transport activity was validated by yeast mutant complementary assay. In addition, the function of the *TaAMT2;3a* in the wheat-*Pst* interaction was analysed using a genetic silencing technique through barley stripe mosaic virus.

## Results

### Determination of ammonium contents in specific tissues of wheat infected by *Pst*

**Fig. 1:**
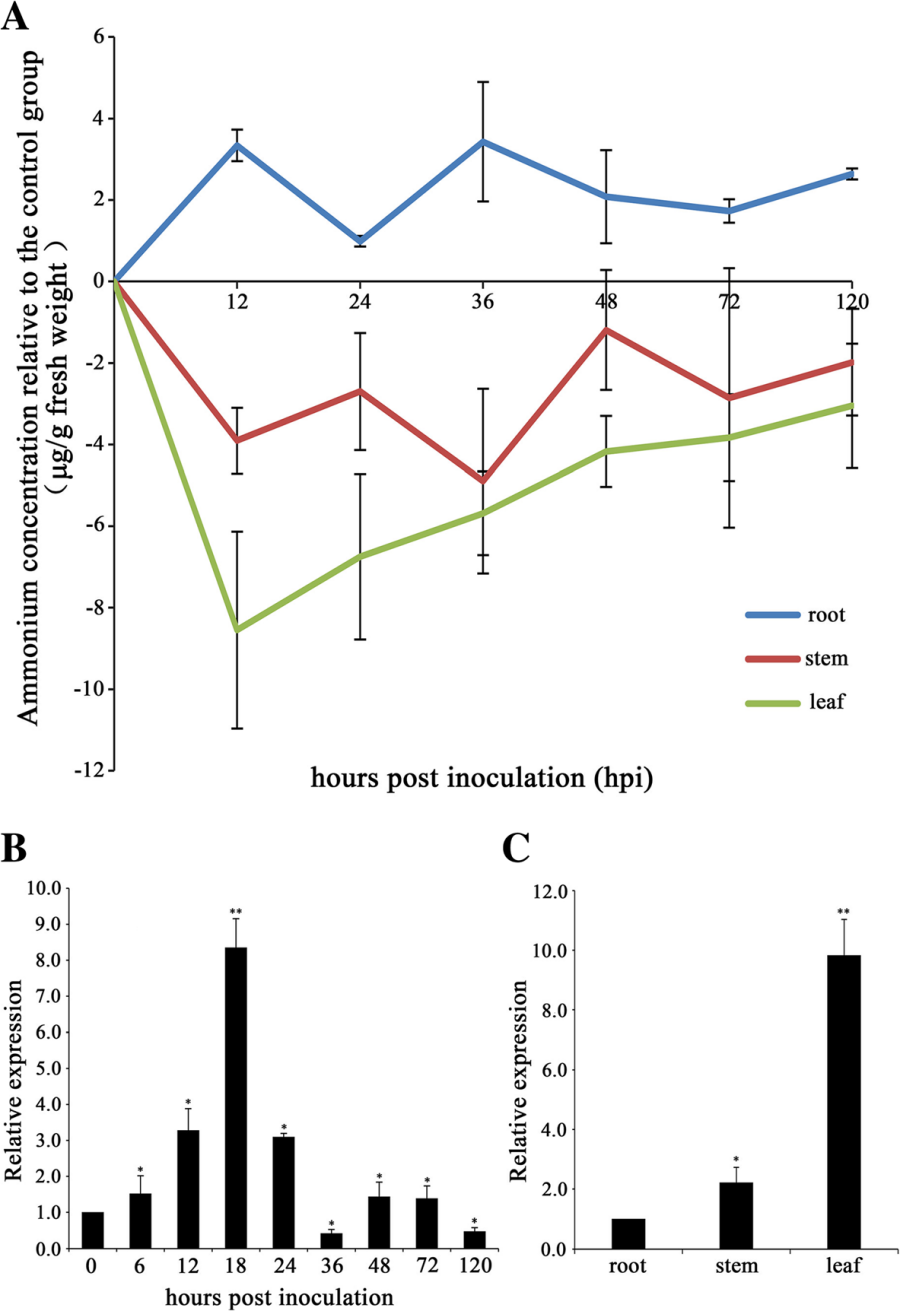
Relative ammonium contents in different wheat tissues after inoculation with *Puccinia striiformis* f.sp. *tritici* (*Pst*) and the expression pattern of *TaAMT2:3a*. **a** Relative ammonium concentrations in leaves, stems, and roots from wheats inoculated with *Pst* at different time points. The relative ammonium concentration was calculated by subtracting the ammonium concentration of the non-inoculated control plants from that of the inoculated plants. **b** Relative transcription levels of the *TaAMT2;3a* gene in wheat leaves inoculated with *Pst* compared with that in the control plants. **c** The expression pattern of the *TaAMT2;3a* gene in different tissues. The data were normalized to the wheat *TaEF-1a* gene. All the results were obtained from three independent replicates. Asterisks indicate significant differences from 0 hpi using Student’s t-test. (*P* < 0.05)

During the interaction between *Pst* and wheat, the pathogen need to uptake nitrogen from the host to support its growth and multiplication. To access the change in ammonium contents in wheat infected by *Pst*, we measured the ammonium concentrations in roots, stems and leaves at 0, 12, 24, 36, 48, 72 and 120 h post inoculation (hpi). The ammonium contents between inoculated wheat and non-inoculated control were compared. The results demonstrated that the relative ammonium contents in the roots increased as early as 12 hpi and were maintained at a high level throughout the infection. In contrast, the relative ammonium contents in the leaves and stems significantly decreased, especially in the leaves, and reached the lowest point at 12 hpi and 36 hpi, respectively (Fig. 1a). These results revealed that the infection by stripe rust fungus greatly affected the transport and metabolism of ammonium in wheat, which led to a decline in ammonium levels in the leaves and stems and an increase in the roots.

### Identification of the wheat AMT2-type AMT gene *TaAMT2;3a* induced by *Pst* infection

The fluctuation of ammonium contents during *Pst* infection might be related to the ammonium transportation. To identify wheat genes involved in ammonium metabolism and transport during *Pst* infection, we screened AMT genes that were differentially expressed during wheat-*Pst* interaction using the transcriptome database [20]. A wheat gene encoding an AMT2-type ammonium transporter was found to be upregulated upon *Pst* infection. This gene was designated as *TaAMT2;3a* according to its homologous gene in *Arabidopsis* and a published reference [12].

The induction of *TaAMT2;3a* by *Pst* infection was further confirmed by quantitative RT-PCR. Analysis of *TaAMT2;3a* expression revealed that during a compatible interaction, the transcription of *TaAMT2;3a* was induced as early as 6 hpi and reached the highest level at 18 hpi, which was 7.3-fold higher than that in the control plants (Fig. 1b). The mRNA levels of *TaAMT2;3a* in different wheat tissues (roots, stems, leaves) were examined. The results indicated that *TaAMT2;3a* was detectable in all tested wheat tissues, and the transcription levels of *TaAMT2;3a* in leaves were significantly higher than those in roots and stems (Fig. 1c). These results suggested that *TaAMT2;3a* may be involved in transporting ammonium in the leaves and play an important role in the wheat-*Pst* interaction.

The full-length CDS (1449 bp) of the *TaAMT2;3a* gene was isolated from wheat variety Suwon11. Sequence alignment with orthologues in the wheat variety Chinese Spring genome from the IWGSC (International Wheat Genome Sequencing Consortium) database showed that there are three homoeologous genes located on chromosomes 1A, 1B and 1D. The *TaAMT2;3a* sequence was identical to the sequence on the long arm of chromosome 1D (Additional file 1: Figure S1).

**Fig. 2:**
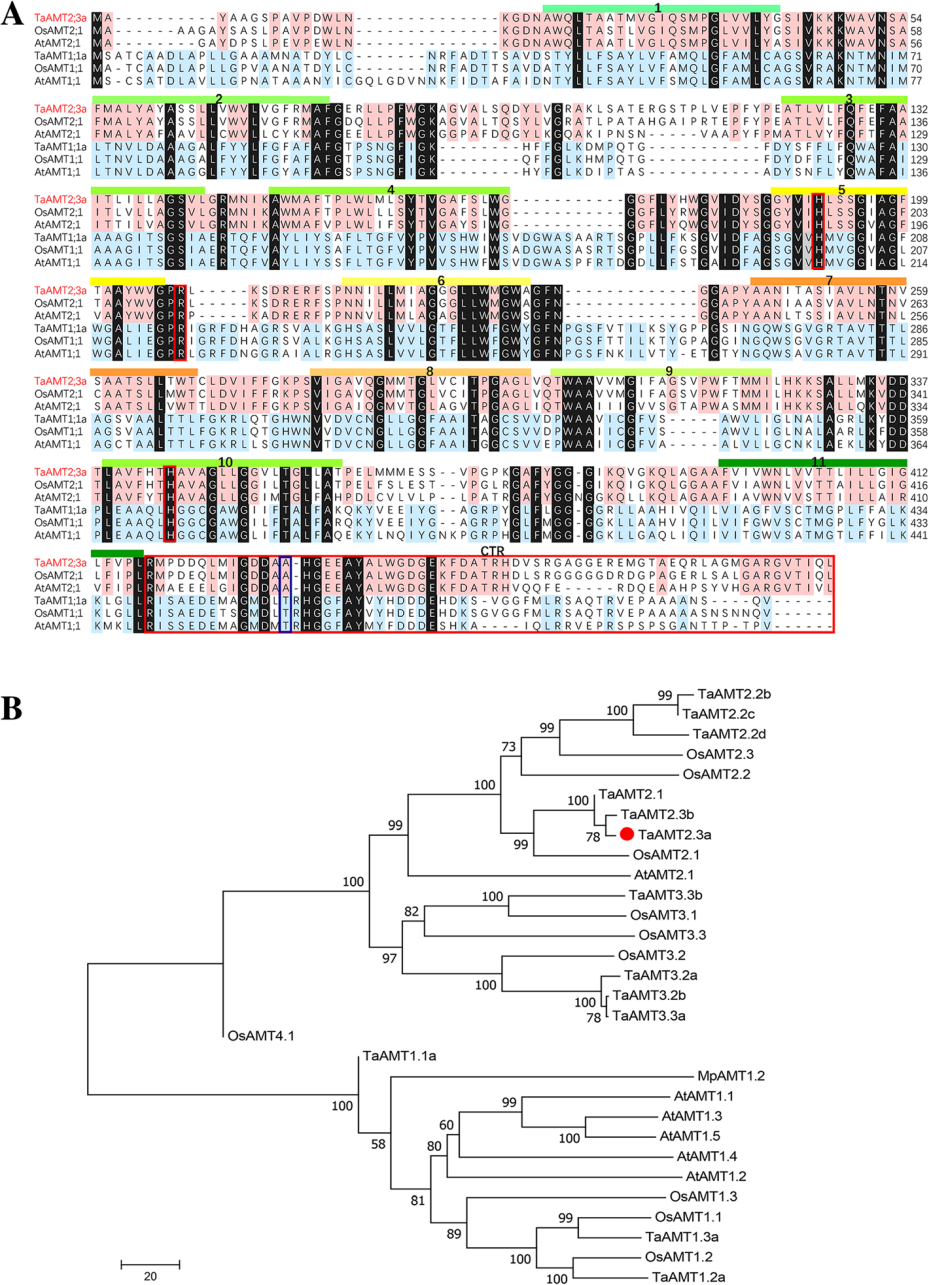
Multiple sequence alignment and phylogenetic analysis of TaAMT2;3a. **a** Multiple sequence alignment of amino acid sequences from *Arabidopsis thaliana* (AtAMT1;1, AtAMT2;1), *Oryza sativa* (OsAMT1;1, OsAMT2;1) and *Triticum aestivum* (TaAMT2;3a)*.* Ammonium transporter domains: red line. CTR: black line. Highly conserved domain: blue linear box. **b** Phylogenetic analysis of TaAMT2;3a and homologues in wheat and other plant species. At: *Arabidopsis thaliana*; Ta: *Triticum aestivum*; Mp: *Marchantia polymorpha*; and Os: *Oryza sativa*. Multiple sequence alignments and the neighbour joining tree were created using the ClustalW algorithm by MEGA (version 7.0)

TaAMT2;3a is a typical ammonium transporter containing a signal peptide, an ammonium transporter region (from 21 to 437 aa) and a C-terminal region (CTR) (Fig. 2a, Additional file 2: Figure S2). Multiple sequence alignment showed that TaAMT2;3a shares the highest identity (83%) with OsAMT2;1 from rice, followed by AtAMT2;1 from *Arabidopsis*, with 71% identity. To better understand the evolutionary relationship between TaAMT2;3a and its homologues from other plant species, a phylogenetic tree was constructed based on the full-length amino acid sequences from wheat, rice, *Arabidopsis* and liverwort (Fig. 2b and Additional file 3: Table S1). Phylogenetic analysis demonstrated that except for OsAMT4;1, other AMTs were unambiguously categorized into two subfamilies, AMT1-type and AMT2-type AMTs. TaAMT2;3a was clustered into the AMT2 group and had the closest evolutionary relationship with OsAMT2.

### TaAMT2;3a is localized to the cytosolic and nuclear membranes in wheat cells and complement yeast ammonium transporter mutant

**Fig. 3:**
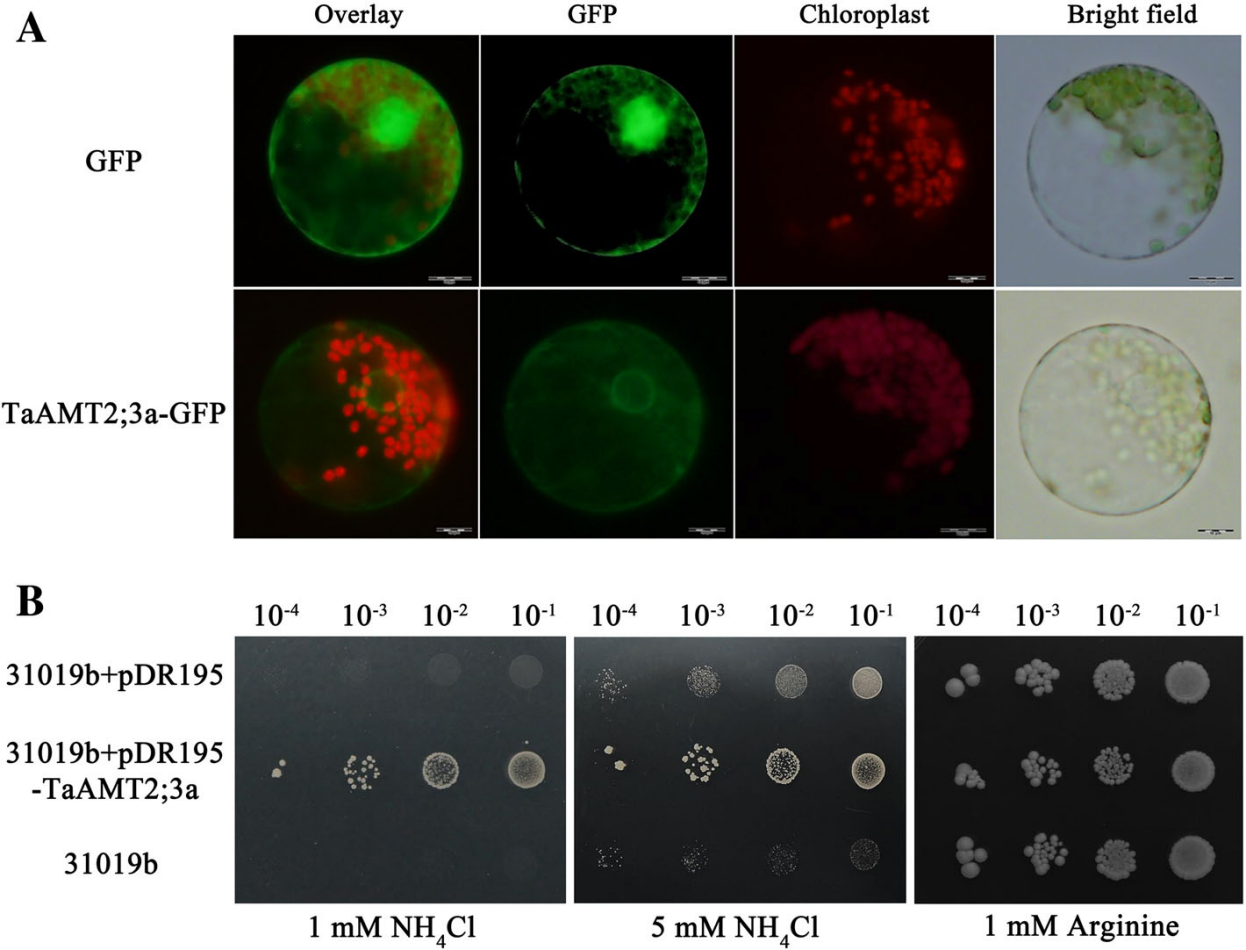
Subcellular localization and functional complementation analysis of TaAMT2;3a. **a** Subcellular localization of TaAMT2;3a in wheat protoplasts. Wheat protoplasts were transformed with a plasmid carrying a eGFP-TaAMT2;3a fusion protein. The picture was obtained using a fluorescence microscope. The top four images indicate GFP (control), and the bottom four images indicate eGFP-TaAMT2;3a. Bar: 10 μm. **b** Functional complementation analysis of TaAMT2;3a in yeast ammonium transporter mutant. Yeast mutant strain 31019b (*mep1*, *mep2*, *mep3*, *ura3*) cells were transformed with a pDR195 vector expressing *TaAMT2;3a* and tested for growth on media supplemented with 1 mM NH_4_Cl, 5 mM NH_4_Cl and 1 mM arginine (Arg). The empty vector (pDR195) was used as the control

Previous studies of AMTs in other organisms have reported that AMTs are mainly located on cell membranes [12, 21]. In the current study, transient expression of the TaAMT2;3a-GFP fusion protein was used to determine the subcellular localization of TaAMT2;3a in wheat protoplasts. Compared with the ubiquitous distribution of GFP in the control group, the fluorescence of the TaAMT2;3a-GFP fusion protein was mainly distributed on the cell and nuclear membranes of wheat cells (Fig. 3a). Thus, the results confirmed that TaAMT2;3a was localized to the cell and nuclear membranes in wheat cells.

To further characterize the biochemical function of TaAMT2;3a, we tested the ability of TaAMT2;3a to transport NH_4_^+^. The TaAMT2;3a protein was expressed in the yeast triple *Mep* deletion strain 31019b (*mep*1, *mep*2, *mep*3, *ura*3), which is entirely defective in NH_4_^+^ transport and unable to grow on medium containing < 5 mM NH_4_^+^ as the sole nitrogen source [21]. The results showed that the yeast mutant transformed with TaAMT2;3a grew on medium containing 1 or 5 mM NH_4_Cl as the sole nitrogen source (Fig. 3b). This finding indicates that TaAMT2;3a complements the NH_4_^+^ uptake defect of 31019b and is likely responsible for high-affinity ammonium transport in plants.

### Silencing of *TaAMT2;3a* impairs fungus pathogenicity during wheat-*Pst* interaction

**Fig. 4:**
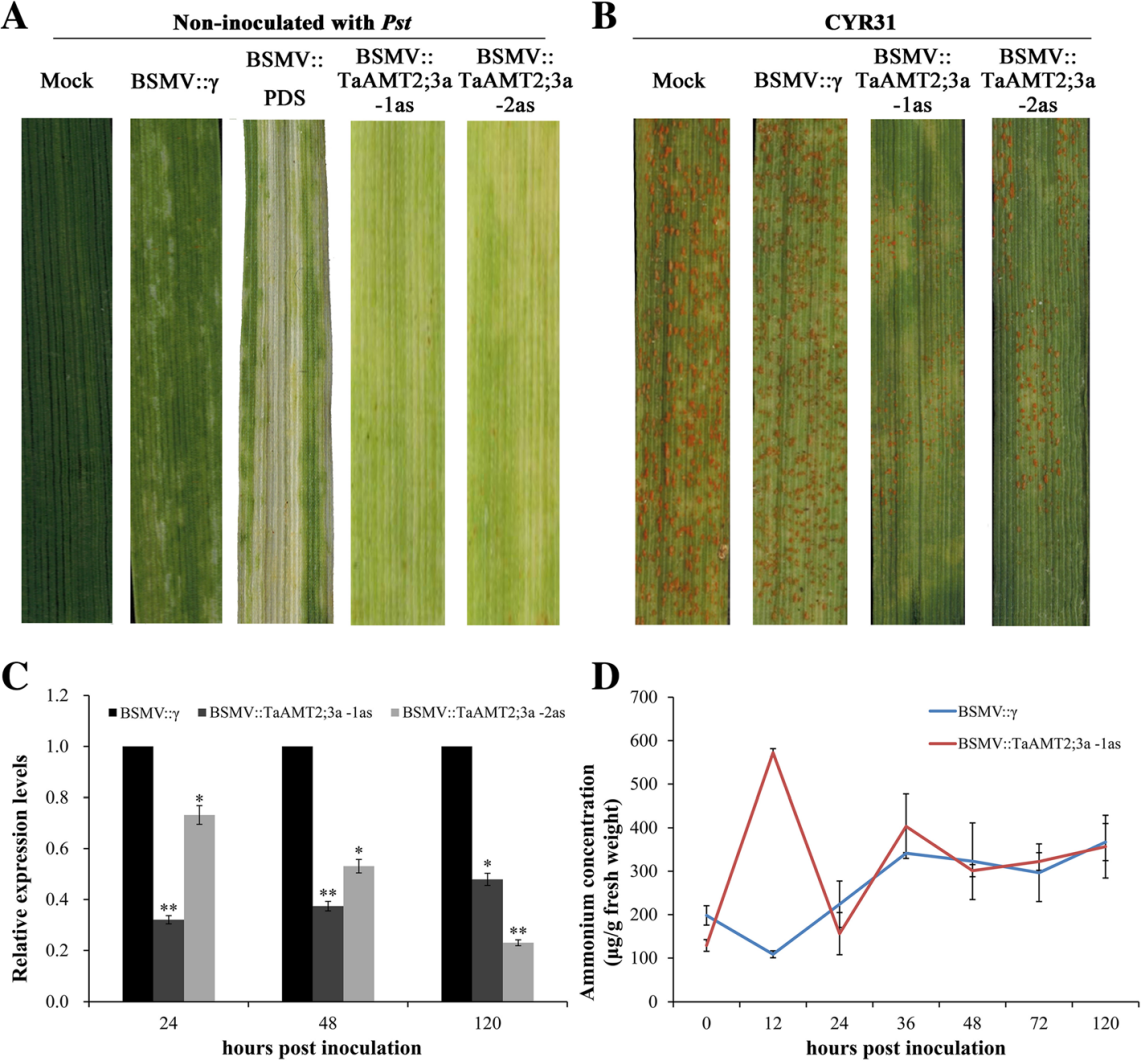
Functional analysis of *TaAMT2;3a* in wheat-*Pst* interaction using a virus-induced gene silencing (VIGS) system. **a** Symptoms of wheat leaves treated by recombinant virus BSMV::γ, BSMV::PDS, BSMV::TaAMT2;3a-1as and BSMV::TaAMT2;3a -2as at 10 dpi. Mock leaves were treated only with FES buffer. **b** The phenotype of the fourth leaves inoculated with *Pst* virulent strain CYR31 at 14 dpi. **c** Relative transcription levels of *TaAMT2;3a*-silencing plants after inoculated with *Pst*. The data were normalized to the *TaEF-1a* expression level. The transcription levels of *TaAMT2;3a* in wheat leaves treated by BSMV::γ at each time point were set to one. (D) Ammonium concentrations in leaves from TaAMT2;3a-knockdown and control wheat plants inoculated with *Pst*. Error bars represent variations among three independent replicates. Asterisks (* and **) indicate a significant differences from 0 hpi using Student’s t-test (*P* < 0.05 and 0.01, respectively)

To reveal the function of the *TaAMT2;3a* gene in the wheat-*Pst* interaction, barley stripe mosaic virus (BSMV)-induced gene silencing (VIGS) was applied. Due to the high similarity among the three *TaAMT2;3a* homoeologs in the wheat A, B, and D genomes, two fragments were designed to silence the three homoeologs together (Additional file 1: Figure S1). The silencing of the wheat phytoene desaturase gene (PDS) was used as the positive control for the gene silencing system to confirm whether the VIGS system functions correctly. Mild chlorotic mosaic symptoms appeared on the fourth leaves in all plants at 10 days after inoculation with BSMV. The BSMV::TaPDS-inoculated plants exhibited strong photobleaching symptoms, suggesting that the VIGS system worked well (Fig. 4a). After inoculation with *Pst*, both *TaAMT2;3a*-knockdown wheat plants (BSMV::TaAMT2;3a -1as and BSMV::TaAMT2;3a -2as) exhibited impaired fungal development compared to the controls (Fig. 4b). Quantitative RT-PCR analyses confirmed the suppression of *TaAMT2;3a* transcription in wheat plants pre-inoculated with virus (Fig. 4c).

The ammonium concentrations of leaves from TaAMT2;3a-knockdown plants as well as those from the control plants were measured. The results showed that the ammonium concentrations in leaves from the TaAMT2;3a-knockdown plants is far more than that from the control plants at 12 hpi, while no apparent difference were observed after 24 hpi (Fig. 4d). These results suggested that suppression of *TaAMT2;3a* gene greatly affected the ammonium transportation at the early stage of *Pst* infection, and retarded the growth of the fungi.

### Histological observation of *Pst* growth in *TaAMT2;3a*-knockdown plants

**Fig. 5:**
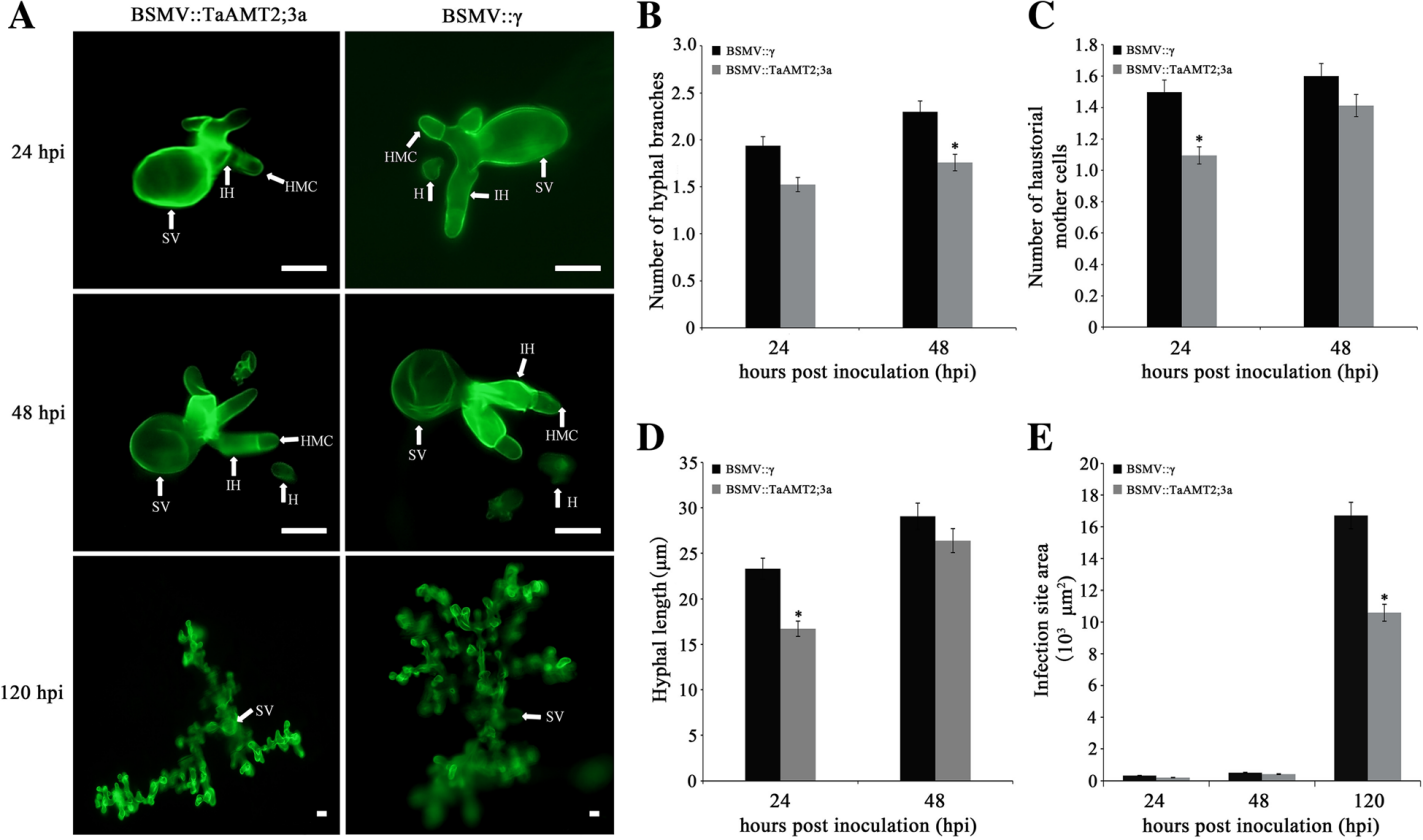
Histological observation of fungi growth in *TaAMT2; 3a*-knockdown plants challenged by *Pst*. **a** Leaves inoculated with *Pst* were observed at 24, 48 and 120 hpi. The infected sites were observed by fluorescence microscopy. H: haustorium; HMC: haustorial mother cell; SV: substomatal vesicle; IH: infection hyphae. Bar, 50 μm; The number of hyphal branches (**b**), the number of haustorial mother cells (**c**), the hyphal length (**d**) and the infection site areas (**e**) in *TaAMT2; 3a*-knockdown plants were compared with that in control plants. The fungal structures were stained with wheat germ agglutinin (WGA) and each result was considered from 30 infection sites. Asterisks indicate significant difference (*P* < 0.05) from BSMV::γ-inoculated plants using Student’s t-test

Leaf samples from *TaAMT2;3a*-knockdown plants were examined by fluorescence microscopy to illustrate fungal development **(**Fig. 5a**)**. As shown in the figure, the number of hyphal branches in wheat leaves from *TaAMT2;3a-*knockdown plants were obviously less than those from control plants at 48 hpi (Fig. 5b**)**. Additionally, the number of haustorial mother cells and the hyphal length at 24 hpi in leaves from *TaAMT2;3a-*knockdown plants were much lower than those from control plants (Fig. 5c and d). For the infection site area, no significant change was observed until 120 hpi (Fig. 5e). Altogether, the overall histological results indicate that suppression of *TaAMT2;3a* expression restricts fungal growth and ultimately affects sporulation and the pathogenicity phenotype.

## Discussion

As a main nitrogen source, ammonium plays an important role in plant growth and development. Knowledge about ammonium uptake and translocation has been obtained by extensive investigations on ammonium transporters in *Arabidopsis* and rice. However, the physiological roles of AMTs in the interaction between plants and environmental microbes remain largely unknown.

Although two types of AMTs share common secondary protein structures and biochemical activities, AMT2-type AMTs showed apparent difference in amino acid sequences, especially in those regions related to transport activity regulation. For example, the T460 residue in *Arabidopsis* AtAMT1 is conserved in all plant AMT1 proteins, and controls ammonium transport activity through phosphorylation and inactivation by the kinase CIPK23 upon extracellular ammonium exposure [22–24]. A phosphomimetic mutation of MpAMT1;2 (T475D) from *Marchantia polymorpha* also exhibited complete loss of ammonium transport activity [25]. With regards to AMT2-type ammonium transporters, an alanine (A) is substituted for the threonine (T) at the corresponding T460 site of AtAMT1;1. Therefore, it seems that AMT2-type AMTs may play a unique role other than ammonium uptake. Recent studies revealed that an AMT2-type ammonium transporter, AtAMT2.1, not only participates in ammonium uptake, but also plays an important role in the translocation of ammonium from the root to the shoot through xylem loading [26]. In the present study, sequence alignment and phylogenetic analysis indicated that TaAMT2;3a is a typical AMT2-type ammonium transporter. In addition, the high expression levels of *TaAMT2;3a* in leaves imply that it is likely involved in ammonium translocation in leaves.

As one of the most important executors of nutrient transport, plant AMTs also play key roles in the interactions between plants and microbes, especially obligate biotrophic fungi. An increasing number of studies have demonstrated that plant AMTs are involved in the mutualistic symbiosis of *Arbuscular mycorrhizae* (AM) fungi. Most of these AMTs belong to the AMT2 subfamily and are highly or exclusively expressed in AM-colonized roots [27]. For example, two *sorghum* AMT2 genes, *SbAMT3;1* and *SbAMT4*, were induced to express in roots colonized by *arbuscular* fungi [28]. *SbAMT3;1* knockdown plants showed greatly reduced uptake of ammonium from the AM network and less growth stimulation by AM fungal colonization [29]. Except for ammonium transport, some AMT members serve as transceptors that sense as well as transport their substrates. In *Medicago* colonized by *Arbuscular mycorrhizae*, AMT2;3 is located in the periarbuscular membrane and is required for the suppression of premature arbuscule degeneration in *pad4* mutants under low-nitrogen conditions [30].

To date, there are only a few reports related to AMT function in plant-pathogen interactions. The *Arabidopsis AMT1.1* mutant displays enhanced resistance against *Plectosphaerella cucumerina* and reduced susceptibility to *Pseudomonas syringae* [31]. Another study demonstrated that three AMT1-type members, *TaAMT1;1a*, *TaAMT1;1b*, and *TaAMT1;3a*, were specifically induced in the compatible interaction between wheat and stem rust fungus (*Puccinia graminis* f. sp. *tritici*, *Pgt*) but not in the incompatible interaction. Additionally, wheat plants grown with NH_4_^+^ were more vulnerable to *Pgt* than plants under N-free conditions, indicating that ammonium transport in root mediated by AMT1-type transporters may facilitate the infection of wheat stem rust [12]. While, the role of AMT2-type transporters in wheat-rust interaction is not clear. In the present study, the increased expression of *TaAMT2;3a* during *Pst* infection was expected to accelerate the ammonium translocation from intercellular space to mesophyll cells. However, the result showed that ammonium concentrations in the aerial part of wheat after *Pst* infection declined, which may be attributed to immediate assimilation of excess ammonium into amino acids [26]. Transcriptional analysis revealed that the single gene encoding AMT in *Pst* was downregulated during wheat infection, while the genes encoding amino acid transporters were upregulated, suggesting that the main form of nitrogen uptake by *Pst* from the host is amino acids rather than ammonium [32]. Thus, *TaAMT2;3a* may be exploited by the rust fungi to meet their demand for nitrogen. This hypothesis was further supported by the retarded fugal growth and increased level of ammonium contents in leaves at the early stage of *Pst* infection in *TaAMT2;3a*-knockdown plants.

This is the first report on the function analysis of AMT2-type AMTs during interactions between plants and pathogens. Our results, together with the previous studies [12], suggest that the coordination between ammonium uptake and translocation mediated by AMT1 and AMT2-type AMTs helps rust pathogens uptake and assimilate nitrogen nutrients from the host. However, how and through what mechanism host ammonium or nitrogen is translocated to the fungus is still unclear. Analysis of other ammonium transporters and a more detailed determination of ammonium dynamics in hosts and pathogens are necessary for a full understanding of the biological roles of plant AMTs in plant-pathogen interactions.

## Conclusions

The results obtained by this study indicate that during the wheat-*Pst* interaction, the wheat AMT2-type gene *TaAMT2;3a* was induced by rust infection and may facilitate the nitrogen uptake by rust fungus. The present study not only provides new knowledge on the function of AMT2-type AMTs in plant-pathogen interaction, but also lays a foundation for revealing the mechanism of nitrogen acquisition from host for rust fungi.

## Methods

### Plant and fungal materials, growth conditions and inoculation

Wheat (*Triticum aestivum*) variety Suwon11 and a Chinese *Pst* race CYR31 (virulent to Suwon11) were obtained from the Prof. Zhensheng Kang’s Lab (Northwest A&F University, China) and were used to study the wheat-*Pst* interaction. Wheat seedlings cultivation and *Pst* inoculation procedures and conditions were followed as described previously [33]. The fresh urediospores of CYR31 were inoculated on the wheat leaves at the first leaf stage. Parallel mock control plants were inoculated with sterile water. After inoculation, the plants were kept in a dark room at 100% humidity for 24 h, then transferred to a growth chamber at 14 °C under a long photoperiod (16 h light/8 h dark). Wheat leaves were sampled at 0, 6, 12, 18, 24, 36, 48, 72 and 120 h post-inoculation (hpi). Meanwhile, samples from leaf, stem and root were collected for gene expression analysis. The time points were selected based on the microscopic study of the wheat-*Pst* interaction [34].

### Cloning of *TaAMT2;3a* and sequence analyses

To clone the TaAMT2;3a gene, primers (Additional file 4: Table S2) was designed based on the sequence from wheat genome sequence using Primer 6.0 software. The amplified fragment of *TaAMT2;3a* CDS from Suwon11 was cloned into the pMD18-T simple vector and sequenced. This cloned sequence was then aligned with the wheat cv. Chinese Spring genome, based on the data of International Wheat Genome Sequencing Consortium (https://urgi.versailles.inra.fr/blast/). The chromosomal locations and related gene sequences were also obtained from this website. Conserved domains were identified using Pfam (http://pfam.xfam.org/) [35, 36]. TMHMM3.0 was used for transmembrane domain prediction [37]. Multiple sequence alignment was performed, and a neighbor joining tree was created using ClustalW and MEGA 7, respectively.

### RNA extraction, cDNA synthesis, and qRT-PCR

Total RNA was extracted with the BiozolTM Reagent (BioFlux, Tokyo, Japan) and treated with DNase I according to the manufacturer’s instructions. Three μg of RNA were subjected to first strand cDNA synthesis with an oligo (dT)_18_ primer using an RT-PCR system (Promega, Madison, WI, USA). Relative quantification of TaAMT2;3a expression was performed with a SYBR Green qRT-PCR mixture on an Biorad CFX Connect Real-Time PCR Detection System (Biorad, USA). Specific primers (Additional file 4: Table S2) were designed and qRT-PCR was conducted according to previously described procedures [38]. The wheat translation elongation factor *TaEF-1a* (GenBank Accession number Q03033) was used as an internal reference for all qRT-PCR assays. All of the reactions were performed in triplicate using independent samples. The comparative 2^–ΔΔCT^ method was used to quantify relative gene expression according to the Threshold values (Ct) [39].

### Subcellular location of TaAMT2:3a-GFP fusion protein

To analyze the subcellular localization of TaAMT2;3a protein, the full length CDS of *TaAMT2;3a* was amplified by primers TaAMT2;3a(163)-F and TaAMT2;3a(163)-R (Additional file 4: Table S2) and inserted into the *Pst*I and *BamH*I sites of the pCAMV35S::GFP vector to generate the TaAMT2;3a-GFP fusion vector. The protoplasts were isolated from leaf tissue of one-week-old wheat seedlings as previously described [40]. The recombinant vector and the control plasmid pCAMV35S::GFP were transformed into wheat protoplasts by the PEG-mediated transformation. The transfected mesophyll protoplasts were incubated in W5 solution in a dark chamber at 23 °C for 18 h. GFP fluorescence were observed with a Zeiss LSM700 confocal laser microscope (Zeiss, Germany) with a 480-nm filter as previously described [41].

### Virus-induced gene silencing (VIGS) of *TaAMT2;3a*

To analysis the function of *TaAMT2;3a* during wheat-*Pst* interaction, virus-induced gene silencing (VIGS) technology was used [42]. Two specific fragments (TaAMT2;3a-1as and TaAMT2;3a-2as) with no similarities to other wheat genes were selected to silence *TaAMT2;3a* (Additional file 4: Table S2). In vitro transcripts were prepared from the linearized plasmids containing the tripartite BSMV genome using the RiboMAX TM Large-Scale RNA Production System-T7 (Promega, Madison, WI, USA) and the Ribom7G Cap Analog (Promega), according to the manufacturer’s instructions. The second leaves from wheat plants in two-leaf stage were infected with BSMV transcripts by rubbing inoculation and then incubated at 25 °C. BSMV: TaPDS was used as a positive control to confirm the successful gene-silencing [42]. Control plants were treated with 1× FES buffer lacking of BSMV transcripts. Virus symptoms of the third leaves were observed and photographed at 10 days post-viral inoculation. The fourth leaves were then inoculated with CYR31 and sampled at 0, 24, 48, and 120 hpi for RNA isolation and histological observation. Wheat phenotypes after *Pst* inoculation were recorded and photographed at 14 dpi. The experiment was repeated three times and 20 plants were used for each fragment each time.

### Histological observations of fungal infection

Wheat leaves infected with BSMV were sampled at 0, 24, 48, and 120 hpi and stained by wheat germ agglutinin (WGA) conjugated to Alexa 488 (Invitrogen, Carlsbad, CA, USA) as previously described [43]. The fungal development in TaAMT2;3a-knockdown plants and control plants infected with *Pst* was observed by microscope. For each sample, at least 30 infection sites from three leaves were examined to assess the number of haustoria and hyphal branches, hyphal length and infection area using Olympus BX53 Digital Fluorescence Microscope and DP-BSW software (Olympus, Tokyo, Japan). Standard deviations and Student’s t-test were applied for statistical analysis.

### Determination of ammonium contents in different tissues of wheat

In order to analyze the levels and change of ammonium nitrogen contents in different tissues from wheat plants infected by *Pst*, wheat seedlings were cultivated by hydroponics method (Hoagland culture medium). Two weeks old wheat seedlings were inoculated with *Pst* race CYR31. Then the leaf, stem and root samples were collected at 7 time points: 0, 12, 24, 36, 48, 72 and 120 h post inoculation (hpi) as well as the non-inoculated control. Leaf samples from BSMV induced gene silencing plants and the control plants were collected at 7 time points the same as above samples. Ammonium was extracted by grinding frozen plant tissue samples into fine powder with a mortar under liquid N2 conditions. The 100 mg tissue powder were then homogenized by 200 μl extraction buffer (50 mm Tris-HCl, pH 8.0, 10 mm imidazole, and 0.5% [w/v] β-mercaptoethanol) in a 1.5 ml microtube. After that, centrifuge the microtube at 12,000 g for 1 min at 4 °C. Transfer the supernatant into a new 1.5 ml microtube and the ammonium concentrations were determined by enzymatic method using an Ammonia Assay Kit (Sigma, Catalog Number: AA0100) according to the manufacturer’s instructions. The relative ammonium concentration was calculated by subtracting the ammonium concentration of the non-inoculated control plants from that of the inoculated plants.

### Yeast complementation assay

The *TaATM2;3a* CDS amplified by primers pDR195-TaAMT2;3a-F and pDR195-TaAMT2;3a-R (Additional file 4: Table S2) was cloned into the *Hind*III site of yeast expression vector pDR195. The ammonium uptake deficient yeast strain 31019b (*mep1*, *mep2*, *mep3*, *ura3*) was obtained from the Bruno Andre (Univ Libre Bruxelles, Belgium). This strain lacks the endogenous ammonium transporters Mep1–3 and therefore is unable to grow on medium containing < 5 mM NH_4_^+^ as the sole nitrogen source [44]. pDR195-TaATM2;3a and empty pDR195 (contains a yeast *Ura3* gene and acts as a negative control) were transformed into 31019b cells. The successful transformants were screened by growing of yeast cells in the SD/−ura solid medium. Positive clones were pre-cultured in liquid yeast in the SD/−ura medium until the absorbance value at 600 nm (OD_600_) reached 0.5. Collect deposits were diluted by 10 times to 10^− 1^, 10^− 2^, 10^− 3^ and 10^− 4^ in 1× Tris-EDTA buffer. Then 6 μL of each transformant was plated on yeast N base media containing 1 mM, 5 mM NH_4_Cl and 1 mM Arginine, respectively. Yeast growth was monitored at 30 °C for 3 days.

## Additional files

Additional file 1: **Figure S1** Sequence alignment of three homoeologs of *TaAMT2;3a* in wheat A, B and D genome. (TIF 12494 kb)

Additional file 2: **Figure S2** The prediction of transmembrane region of wheat TaAMT2;3a by TMHMM3.0. (TIF 159 kb)

Additional file 3: **Table S1.** List of names, accession ID and protein sequences of ammonium transporters genes analyzed in this study. (XLSX 15 kb)

Additional file 4: **Table S2** Information of primers used in this study. (XLSX 10 kb)

## Data Availability

Data generated or analyzed in this study are included in this article and its supplementary information files.

## Abbreviations

AM: *Arbuscular mycorrhizal*
AMT: Ammonium transporter
Arg: Arginine
At: *Arabidopsis thaliana*
bp: Base-pairs
BSMV: Barley stripe mosaic virus
EHM: Extrahaustoria matrix
H: Haustorium
HMC: Haustorial mother cell
hpi: Hours post-inoculation
IH: Infection hyphae
Mp: *Marchantia polymorpha*
NIS: Nitrogen-induced susceptibility.
NRT: Nitrate transporter
ORF: Open reading frame
Os: *Oryza sativa*
PDS: Phytoene desaturase gene
*Pgt*: *Puccinia graminis* f. sp. *tritici*
*Pst*: *Puccinia striiformis* f. sp. *tritici*
qRT-PCR: Quantitative realtime-PCR
SV: Substomatal vesicle
Ta: *Triticum aestivum*
VIGS: Virus-induced gene silencing
WGA: Wheat germ agglutinin
