# Supplementary figures and images for: TaAMT2;3a, a wheat AMT2-type ammonium transporter, facilitates the infection of stripe rust fungus on wheat
Source: BMC Plant Biol. 2019 Jun 6;19:239. doi: 10.1186/s12870-019-1841-8 (PMC6554902; doi:10.1186/s12870-019-1841-8)

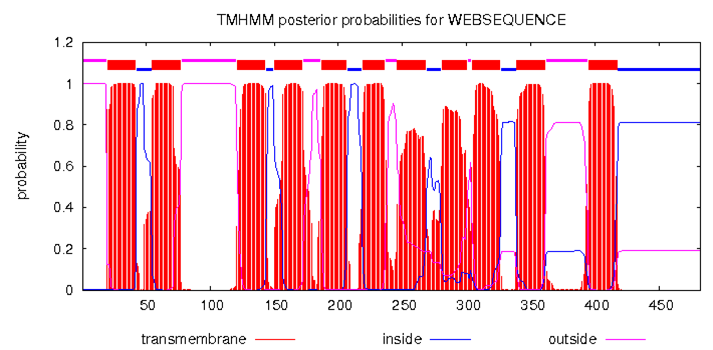

Supplement: Supplementary file 2 — Figure S2 The prediction of transmembrane region of wheat TaAMT2;3a by TMHMM3.0. (TIF 159 kb) [file 12870_2019_1841_MOESM2_ESM.tif]
